# Food-Borne Viruses: Progress and Challenges

**DOI:** 10.3201/eid1411.080819

**Published:** 2008-11

**Authors:** Mette Myrmel

**Affiliations:** Norwegian School of Veterinary Science, Oslo, Norway

One question that is well known to persons working with food virology, and especially noroviruses, is “How important are these viruses, actually?” Frequently you feel somewhat uneasy when you start to reply, talking about the “trivial illness” of gastroenteritis, continuing on to the economic impact caused by the huge number of cases, and finally ending up admitting that you really do not know the answer. This book does not provide an answer to this difficult question either, but it does give you, among other things, an introduction to the pitfalls in trying to “estimate the burden of an underreported disease.” One conclusion is that we probably underestimate its importance.

The book consists of 10 chapters written by persons well known to food virologists as experts in their fields. It is well organized, well written, and presents the history of foodborne viruses, the state of the art, and a glance into the future. The history of food virology is given by Dean O. Cliver, one of the pioneers in this area, who spices up his contribution by sharing interesting personal experiences with the reader. Most of the focus in the book is, naturally, directed toward norovirus and hepatitis A virus. These viruses are covered by up-to-date presentations on molecular biology, clinical disease and diagnostics, pathogenesis and immunity, epidemiology, and detection in food matrixes. Recommendations are also given about which foods should be tested and under which circumstances. Although protocols are not presented, many references are listed.

The molecular revolution has improved the detection of viruses that are impossible or difficult to propagate in cell culture, such as norovirus and hepatitis A virus, and reverse transcription–PCR has become the standard method for virus detection in food. However, sensitive detection methods are still lacking. This is one of the recurrent topics in the book because it affects outbreak investigation, risk assessment, and risk management.

The need for standardization for virus detection in food is stated, as is the proper use of controls. Nobody will argue against detection of false-negative and false-positive results, but some thoughts on the cost-benefit aspects regarding accurate quantification of virus genomes in foods would have been interesting.

The book provides a broad overview of the present situation and gives a good introduction to the topic. Factors that may contribute to the emergence of new viral diseases are also discussed, including demographic and pathogen-related changes. This is interesting reading because virus families and viral evolution are discussed within the context of foodborne transmission. The editors encourage the reader to enjoy the book, and, most of the time, I did.

